# Update on cutaneous mesenchymal tumors in the 5th edition of WHO classification of skin tumors with an emphasis on new fusion-associated neoplasms

**DOI:** 10.1007/s00428-024-03925-2

**Published:** 2024-09-12

**Authors:** Antonina V. Kalmykova, Vira Baranovska-Andrigo, Michael Michal

**Affiliations:** 1 Medical Laboratory CSD, Ltd., Kiev, Ukraine; 2 Department of Pathology, Charles University, Faculty of Medicine in Plzen, Medical Faculty and Charles University Hospital Plzen, Alej Svobody 80, 323 00 Plzen, Czech Republic; 3 Bioptical Laboratory, Ltd., Pilsen, Czech Republic

**Keywords:** *MITF* pathway–activated melanocytic tumors, *CRTC1::TRIM11* cutaneous tumor, *EWSR1::SMAD3*-rearranged fibroblastic tumor, *NTRK*-rearranged spindle cell neoplasm, Superficial CD34+ fibroblastic tumor, *ACTIN::MITF*, *MITF::CREM* fusion

## Abstract

The section on mesenchymal tumors in the 5th edition of WHO classification of skin tumors has undergone several changes, the most important of which is the inclusion of newly identified tumor entities, which will be the main focus of this review article. These specifically include three novel cutaneous mesenchymal tumors with melanocytic differentiation, and rearrangements of the *CRTC1::TRIM11*, *ACTIN::MITF*, and *MITF::CREM* genes as well as *EWSR1::SMAD3*-rearranged fibroblastic tumors, superficial CD34-positive fibroblastic tumors, and *NTRK*-rearranged spindle cell neoplasms. Some of the other most important changes will be briefly mentioned as well.

## Introduction

Published in 2023, the 5th edition of the WHO classification of skin tumors has undergone some significant changes in the mesenchymal tumors section. The main focus of this review will be the novel tumor entities included in this latest edition while the inclusion of other selected changes, such as alterations in terminology and novel (mainly molecular) findings added to established entities will be only briefly mentioned. All novel tumor entities, their salient clinicopathological and molecular features, and key differential diagnostic considerations are summarized in Table 1.

**Table 1:** Characteristic clinicopathological and molecular features of novel WHO entities

Entity	Clinical features	Pathological features	Immunohistochemical features	Molecular features	Prognosis	Differential diagnosis
*CRTC1::TRIM11* tumor	- 40< reported cases - Distal extremities and trunk - Well-defined papule or nodule - Rarely observed in submucosal regions	- Dermal nodule with occasional extension into subcutis - Short intersecting fascicles or nests of epithelioid to spindle cells - Uniform cells with moderate to severe atypia, prominent nucleoli and eosinophilic cytoplasm - Limited mitotic activity (< 5 mitoses/10 HPF), no atypical forms	- SOX10+, S100 (≈ 80%, often focal/patchy) - variable HMB-45 and Melan A (at least one of them positive in ≈80%)	*CRTC1::TRIM11* fusion	- 6/38 cases with adverse events - LR (3/6), LN met (5/6), pulmonary met (3/6)	- Cellular blue nevus - Melanoma - Clear cell sarcoma - PEComa - *MITF::CREM* tumors
*MITF::CREM* tumor	- 3 reported cases: - 2 newborns (one F, one of unknown gender), 1 adult F - Locations: Scalp (*n=*2), hand (*n=*1)	- Dermal nodule with some subcutaneous extension - Infiltrative growth - Two cases abutting the epidermis but no intraepidermal component - Solid sheets, small nests, or bundles of epithelioid to spindle cells with moderate atypia, prominent nucleoli - Eosinophilic or clear cytoplasm - Variable mitotic activity (4-11 mitoses/10 HPF), occasional atypical forms	- 3/3 tested cases: S100, SOX10 positive - 2/2: MITF and HMB-45 positive - Melan A: 2/3 cases positive	*MITF::CREM* fusion	- 1/3 case recurred locally at 9m, NED 23m later - 2/3 cases NED 9 and at 39m	- Cellular blue nevus - Melanoma - Clear cell sarcoma - PEComa - *CRTC1::TRIM11* tumors
*ACTIN::MITF* tumor	- 9 reported cases (+1 in our files) - All F aged 15-75 years (median 40 years) - Extremities (7/10 cases), breast (1/10)	- Infiltrative dermal lesion with subcutaneous extension - Solid sheets of large epithelioid cells with clear cytoplasm, mild to moderate atypia and variably prominent nucleoli - Characteristic dissection of collagen bundles (checkerboard-like pattern) - Minimal mitotic activity (0-1 mitosis/10 HPF)	- S100 protein, tyrosinase, HMB-45, Melan A: variable positivity - Some cases negative for all these markers - 2/2 cases: SOX10 negative	*- ACTB::MITF* - *ACTG1::MITF* fusions	NED 3/3 cases (follow-up: 7-17 years)	- PEComa - Cellular blue nevus - Melanoma - Clear cell sarcoma
*EWSR1::SMAD3* rearranged fibroblastic tumor	- 17 documented cases - All age groups - F>M - Acral location (lower>upper limbs) - one bone case	- Dermal lesion with subcutaneous extension - Densely packed cellular bundles of monomorphic bland spindle cells - Prominently hyalinized stroma - Mitotic activity low or absent	- ERG+ - Keratins, CD34, CD31: negative - SMA occasionally weakly positive	*EWSR1::SMAD3* fusion	Favorable (recurs if incompletely excised)	- Cellular FH - Myofibroma - Fibromatosis - Monophasic synovial sarcoma - DFSP - Low-grade fibromyxoid sarcoma
Superficial CD34+ fibroblastic tumor	- Adolescents or young adults - Proximal limbs (particularly thigh)	- Well-circumscribed dermal/subcutaneous tumor - Sheets/bundles of epithelioid/spindle cells with marked pleomorphism and frequent nuclear pseudoinclusions - Abundant, markedly eosinophilic, granular to glassy cytoplasm - Usually low mitotic activity and lack of atypical mitoses	- All cases: CD34 and SynCam3 positivity - ≈ 70-80%: focal/patchy keratin expression - ≈ 2/3 of cases: N-terminus WT-1 positivity	*PRDM10* fusions with *CITED2*, *MED12*, *ARHGAP32* or *RAD30* genes	- Indolent - Rare LR and LN mets	- UPS - Pleomorphic liposarcoma - DFSP - Myxoinflammatory fibroblastic sarcoma - Atypical cellular FH
*NTRK*-rearranged spindle cell neoplasms	-Children>adults - Any location	- Continuous spectrum of spindle cell tumors that based on cellularity, degree of atypia and mitotic activity can be divided into low, intermediate and high-grade lesions	- Common co-expression of CD34, S100 protein and CD30+, - PanTRK and ALK positive in cases with respective fusions	*NTRK1/2/3, BRAF, RAF1, RET, ALK, MET*, rarely other kinase fusions - *BRAF, EGFR* mutations	- LR: 7-26% cases - mets: 5-20% - high-grade tumors more aggressive	*- LG group:* Lipofibromatosis DFSP SFT Benign neurogenic tumors *- HG group:* MPNST Fibrosarcoma arising in DFSP

*DFSP*, dermatofibrosarcoma protuberans; *F*, female; *FH*, fibrous histiocytoma; *m*, months; *M*, male; *MPNST*, malignant peripheral nerve sheath tumor; *LN*, lymph node; *LR*, local recurrence; *SFT*, solitary fibrous tumor; *UP*S, Undifferentiated pleomorphic sarcoma

## *MITF* pathway–activated cutaneous melanocytic tumors (tumors with *ACTIN::MITF, MITF::CREM*, and *CRTC1::TRIM11* fusions)

This group comprises three recently identified tumor types characterized by distinct genetic alterations: tumors with *CRTC1::TRIM11*, *ACTIN::MITF*, and *MITF::CREM* fusions [1–8]. However, in the WHO classification, these tumors were placed in different chapters. *ACTIN::MITF-* and *MITF::CREM*-rearranged tumors are categorized as melanocytomas in the melanocytic tumors chapter while neoplasms with *CRTC1::TRIM11* fusions are classified as malignant soft tissue tumors with uncertain differentiation. Notably, in contrast to the highly malignant nature of clear cell sarcoma, i.e., a tumor in many aspects similar to these three novel tumors, the latter exhibit a more favorable prognosis [1, 3–6]. Despite shared pathogenesis, some of their clinicopathological features differ significantly. Nevertheless, there are also several recurrent features shared by all three tumor types.

All three tumors tend to involve the dermis, with variable extension into the subcutis, and typically lack an intraepidermal component. Additionally, they exhibit a similar immunophenotype characterized by frequent, albeit variable, expression of HMB-45 and Melan A (most cases show positivity with at least one of these markers). Notably, tumors with *CRTC1::TRIM11* and *MITF::CREM* fusions consistently demonstrate diffuse expression of SOX10 and typically express S100 protein, while *ACTIN::MITF* tumors display variable S100 protein expression and are usually negative for SOX10 (although only two cases were tested) [1–8].

The tumors share similar morphology and immunophenotype, leading to a common differential diagnosis, including cellular blue nevus, melanoma, clear cell sarcoma, and PEComa. *CRTC1::TRIM11* and *MITF::CREM* tumors have identical differential diagnoses and thus will be discussed only in the section on *CRTC1::TRIM11* tumors. *ACTIN::MITF* tumors also have a similar differential with the previously mentioned entities but due to a slightly different immunophenotype, it warrants a separate discussion.

While the specific molecular mechanisms vary to some extent and have been previously explained in detail [4, 5], they ultimately lead to the overexpression of MITF in both *CRTC1::TRIM11* and *ACTIN::MITF*, as well as *MITF::CREM*-associated tumors. MITF, part of the *MIT/TFE* gene family alongside *TFEB*, *TFE3*, and *TFEC* genes, serves as a key regulatory protein in the development of melanocyte lineage. It not only regulates the expression of melanocyte proteins, such as HMB-45, but it also supports melanocyte survival and proliferation, implying its potential involvement in tumorigenesis [4, 9]. In melanocytes, its expression is triggered physiologically by a signaling pathway activated by melanocyte-stimulating hormone (MSH). Elevated MSH level increases cAMP, activating cAMP-dependent transcription factors from the CREB family, which comprises CREB1, ATF1, and CREM. These proteins directly recognize CREB in the *MITF* promoter, impacting MITF expression [1, 3, 4, 10, 11]. Interestingly, all three discussed fusions seem to activate this signaling pathway at various levels. *ACTIN::MITF* rearrangement results in MITF overexpression most likely by directly impacting this protein [4]. In contrast, similar to *EWSR1::ATF1* fusion in clear cell sarcoma, both *CRTC1::TRIM11* and *MITF::CREM* fusions likely lead to MITF overexpression through the induction of the CREB signaling cascade [1, 3, 5, 6]. However, it is unclear at this point whether melanocytic differentiation of these tumors (i.e., expression of S100, SOX10, HMB-45, Melan A, etc.) is attained via origin in a soft tissue melanocyte or via their distinct genetic alterations resulting in the activation of the MITF pathway or both [12]. Nevertheless, some of the experts in this field seem to favor the latter possibility which appears as a much more logical explanation to us as well [12].

## *CRTC1::TRIM11* cutaneous tumor

**Fig. 1:**
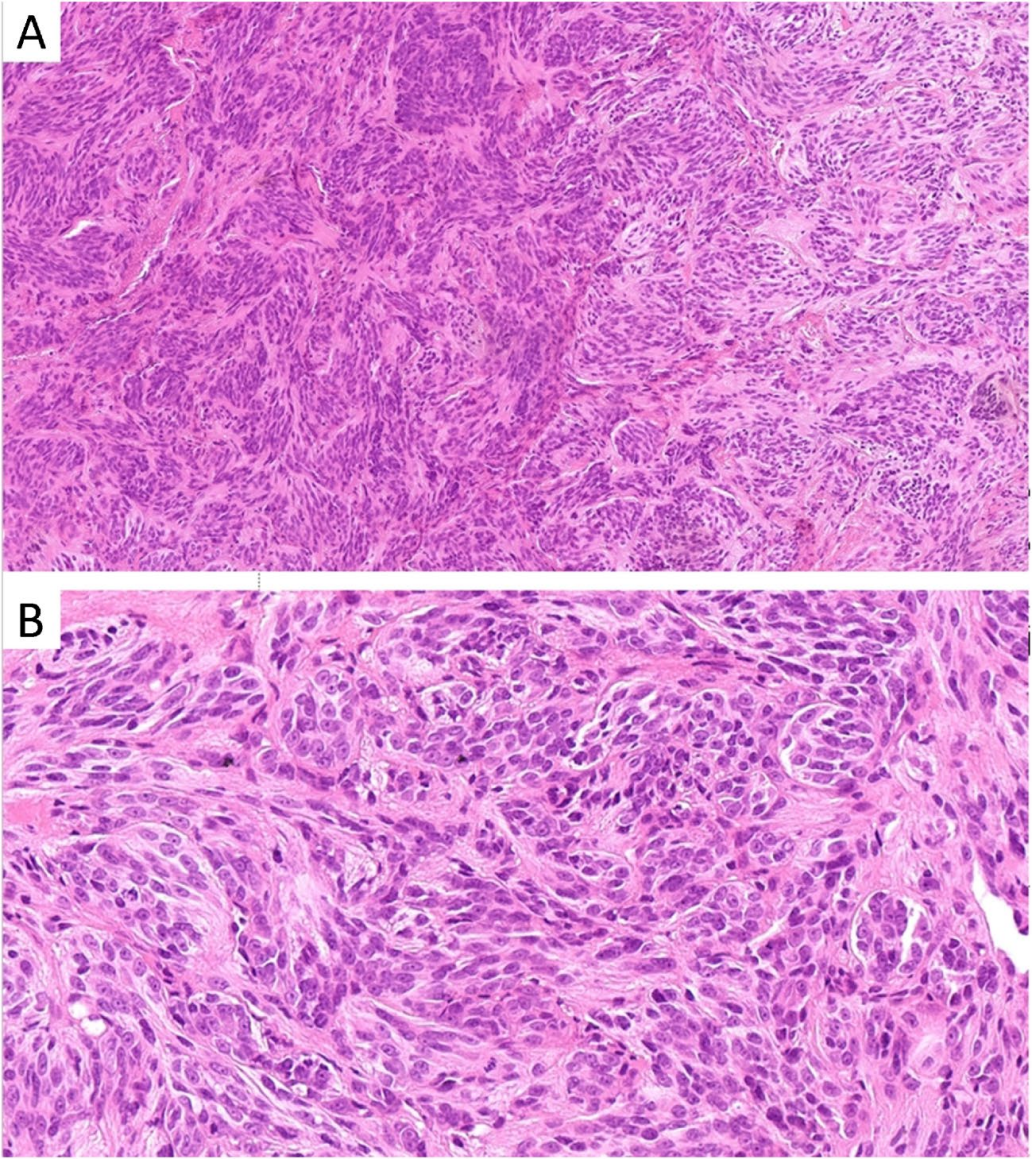
**A**,** B** *CRTC1::TRIM11*-rearranged tumor is usually composed of short intersecting fascicles or nests of epithelioid to spindle cells with eosinophilic cytoplasm, prominent nucleoli, and minimal mitotic activity (< 5 mitoses/10 HPF), without atypical forms

The subgroup characterized by *CRTC1::TRIM11* fusion is relatively well-documented, with over 40 reported cases so far [2, 5, 7, 8]. Typically found on the distal extremities and trunk, this tumor presents as a well-defined dermal nodule, occasionally extending into subcutis. Almost all cases lacked an intraepidermal component apart from one case which, most likely as a result of an upward growth of a primarily dermal tumor, exhibited junctional nests of tumor cells, similar to what has been described in rare cases of clear cell sarcoma [13–15]. Individual cases have been observed in submucosal regions [5]. The tumor consists of short intersecting fascicles or nests of relatively uniform epithelioid to spindle cells with eosinophilic cytoplasm, moderate to severe atypia, prominent nucleoli, and limited mitotic activity (< 5 mitoses/10 HPF), without atypical forms (Fig. 1). Binucleated or multinucleated cells are occasionally observed, yet wreath-like multinucleated giant cells, which can be observed in clear cell sarcoma, are absent. Small areas of necrosis are infrequently detected. Immunohistochemically, the tumor consistently exhibits positivity for SOX10 and in approximately 80% of cases demonstrates expression of S100 protein, albeit often limited in extent. Furthermore, at least 60% of reported cases show positivity for HMB-45 and Melan A, with at least one of these markers being positive in 80% of cases [2, 5, 7].

As previously mentioned, the main entities in the differential diagnosis include cellular blue nevus, melanoma, clear cell sarcoma, and PEComa. Cellular blue nevus typically presents with heavy pigmentation and often features a conventional blue nevus component at the periphery of the lesion. Mutations in *GNAQ* or *GNA11* confirm the latter diagnosis in challenging cases. Cutaneous melanoma commonly exhibits an intraepidermal component, along with significantly higher pleomorphism and mitotic activity, including common atypical forms. The most frequently observed mutations in melanomas include *BRAF*, *NRAS*, *NF1*, etc. Moreover, genetic alterations such as a high mutational burden and a UV signature are another characteristic features of melanoma [16]. In both *CRTC1::TRIM11* and *MITF::CREM* tumors, clear cell sarcoma poses the most challenging differential diagnosis due to their striking similarities. Clear cell sarcoma, typically localized around the ankle, may be distinguished by the presence of multiple wreath-like multinucleated giant cells. However, accurate differentiation often requires molecular methods. Detection of *EWSR1* gene rearrangement and/or confirmation of an *EWSR1::ATF1* or *EWSR1::CREB1* gene fusion confirms the diagnosis of clear cell sarcoma. Distinguishing *CRTC1::TRIM11* and *MITF::CREM* tumors from PEComa can be easily achieved by SOX10 staining, which is negative in the latter [1, 3, 5, 6].

The prognosis of these tumors is relatively favorable. However, out of 38 cases with follow-up information, 6 showed an adverse event either in the form of a local recurrence (*n* = 3/6), lymph node metastasis (5/6, unconfirmed histologically in one case), or pulmonary metastasis (3/6, unconfirmed histologically in one case) [5, 8, 17–19].

Finally, a very recent study reported three cases with a novel *MED15::ATF1* fusion. Briefly, all three patients were children (5–16 years old) and the tumors occurred on the arm, cheek, and scalp. All were centered to the dermis but one of the cases showed epidermotropism as well. Most of their morphological and immunohistochemical features were otherwise similar to *CRTC1::TRIM11* tumors. Despite limited follow-up, one of the cases showed an early spread to regional lymph nodes [12].

## Tumors with *MITF::CREM* fusion

This group of tumors represents a so far understudied subset, with only three documented cases reported in the literature, one of which was reported by our institution [1, 3, 6]. Among these cases, two were identified in newborns (one female and one of unknown gender), while the third occurred in a 37-year-old woman. Tumor locations varied, with two cases affecting the scalp and the remaining case arising on the dorsum of the hand. Given the limited available data, the prognosis of these tumors is not well established. From the three reported cases, one recurred locally 9 months after an incomplete excision and the patient had no evidence of disease 23 months later. The other two reported cases had no evidence of disease 9 and 39 months after complete excision.

**Fig. 2:**
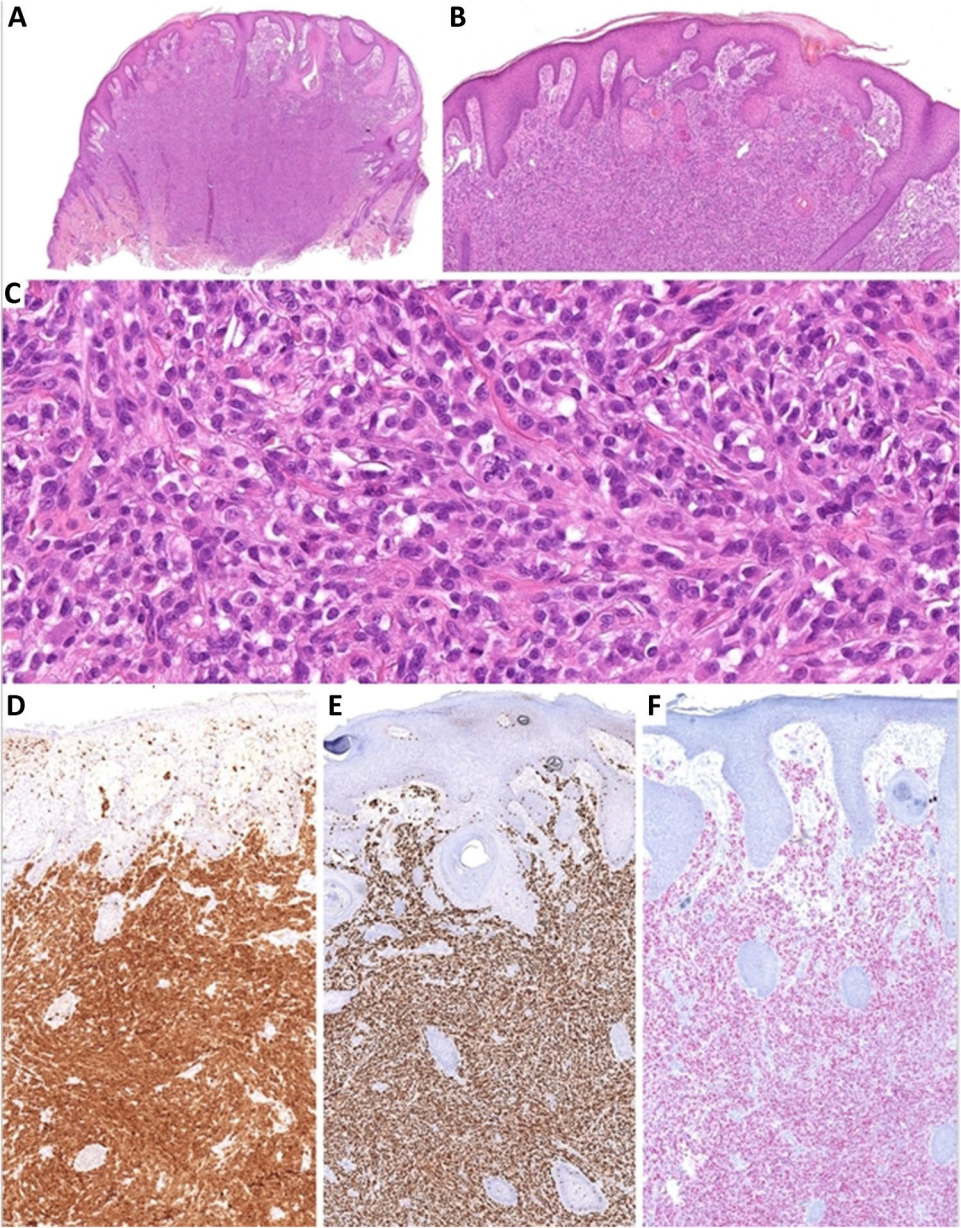
**A** *MITF::CREM*-rearranged tumor shows infiltrative growth patterns, lacking well-defined margins, and predominantly involves the dermis sometimes with subcutaneous extension.** B** Despite the close proximity to the epidermis, no intraepidermal component is present.** C** All documented lesions consisted of highly cellular proliferation of epithelioid to spindle cells arranged in small solid nests or bundles, particularly evident at the periphery. One case predominantly displayed cells with pale cytoplasm, while the remaining two cases consisted of cells with predominantly eosinophilic cytoplasm (shown here). Tumor cells demonstrated moderate to marked nuclear atypia, prominent nucleoli, and increased mitotic activity including atypical mitoses (center). S100 (**D**), SOX10 (**E**), and MITF (**F**) positivity was detected in all tested cases

Histology of all tumors exhibited infiltrative growth patterns, lacking well-defined margins, predominantly involving the dermis with partial subcutaneous extension (Fig. 2A). Although two cases closely abutted the epidermis, no intraepidermal component was noticed (Fig. 2A, B). All documented lesions consisted of highly cellular proliferation of epithelioid to spindle cells arranged in solid sheets or small solid nests or bundles which were particularly evident at the periphery. While clear cells predominated in one case, the remaining two cases consisted of cells with predominantly eosinophilic cytoplasm. Tumor cells demonstrated moderate nuclear atypia, prominent nucleoli, and increased mitotic activity (ranging from 4 to 11 mitoses/10 HPF), including atypical forms (Fig. 2C). Similar to *CRTC1::TRIM11* tumors, diffuse strong expression of S100 protein (Fig. 2D), SOX10 (Fig. 2E), and MITF (Fig. 2F) was observed in all tested cases, with HMB-45 and Melan A positivity detected in two out of three cases. The differential diagnosis closely mirrors that of *CRTC1::TRIM11* tumors; however, distinguishing between the two groups is very challenging [1, 3, 6]. Morphologically, the primary distinction often lies in the sharper demarcation and more fascicular arrangement of cells in *CRTC1::TRIM11* tumors. Nonetheless, molecular methods may be indispensable for a reliable distinction.

## Tumors with *ACTIN::MITF* fusions (*ACTB::MITF* OR *ACTG1::MITF* fusions)

Limited information for this tumor subtype is available, derived from a single publication that included seven cases [4] and one additional very recent study primarily focusing on PEComas [20]. Nonetheless, the clinicopathological characteristics of these tumors appear distinct and were also observed in one additional case identified at our institution. Among the eight cases with clinical information known to us to date, all occurred in women of different ages (15–75 years, median 40 years), with seven affecting the extremities and one observed on the breast. Notably, no recurrence or metastasis were documented among the three cases with available clinical data, with follow-up durations ranging from 7 to 17 years [4].

**Fig. 3:**
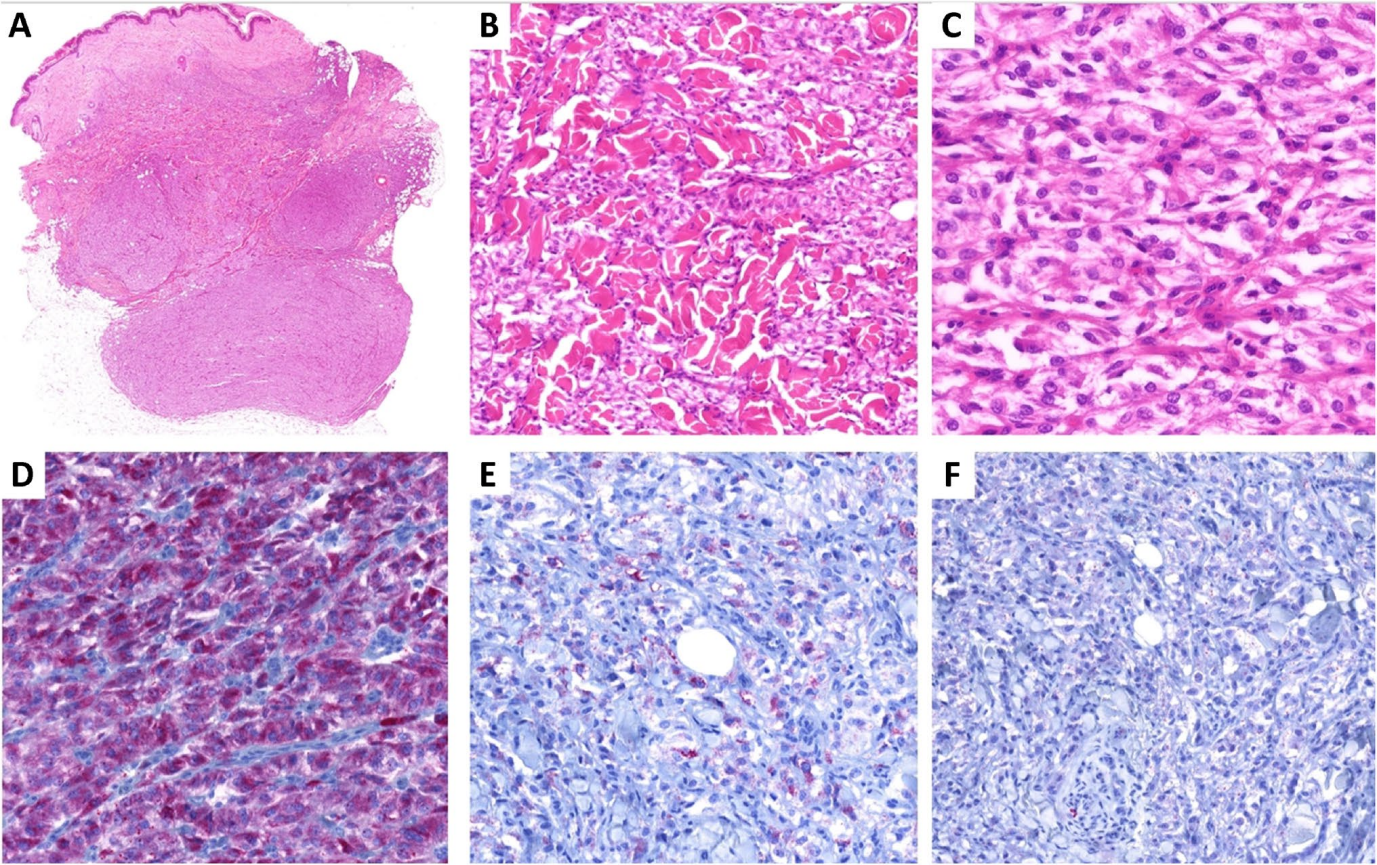
**A** *ACTIN::MITF*-rearranged tumor manifests as dermal lesions with significant infiltrative growth pattern often extending into the subcutis.** B** It consists of solid sheets of bulky epithelioid cells with bright eosinophilic cytoplasm, intermingled between bundles of collagen (so-called checkerboard-like pattern of growth).** C** Cytomorphology is characterized by mild to moderate atypia, variably prominent nucleoli, and minimal mitotic activity. The tumor is typically positive with melanocytic markers such as tyrosinase (**D**) or HMB45 (**E**) although the expression may be focal.** F** Two tested cases were negative with SOX10

This tumor typically manifests as a dermal lesion which, due to a highly infiltrative growth pattern, often extends into the subcutis (Fig. 3A). It consists of solid sheets of large epithelioid cells with clear cytoplasm which characteristically dissect the surrounding collagen bundles (so-called checkerboard-like pattern of growth [20]) (Fig. 3B). Cytomorphology is characterized by mild to moderate atypia, variably prominent nucleoli, and minimal mitotic activity (0–1 mitosis/10HPF) (Fig. 3C). Immunohistochemically, variable expression of S100 protein, tyrosinase (Fig. 3D), HMB-45 (Fig. 3E), or Melan A is commonly present, although some cases were also completely negative for all these markers. SOX10 was negative in one case published [4] as well as in our case (Fig. 3F). Regarding the genetic background, this tumor is associated with the alteration of *MITF* gene, which is fused either with the *ACTB* or *ACTG1* gene [4]. Both of these genes belong to the *ACTIN* family and so the fusions observed are collectively referred to as *ACTIN:MITF* in the literature.

Thanks to their characteristic morphology and minimal mitotic activity, this tumor can be reliably differentiated from blue nevus, clear cell sarcoma, and melanoma. However, distinguishing it from PEComa is more challenging and in some cases impossible without the use of molecular methods. Typical cases with clear cell morphology may be difficult to distinguish particularly from the *TFE3*-rearranged PEComa subtype, which is composed of similar looking clear cells and has a similar immunophenotype (*TFE3* gene belongs to the same *MIT/TFE* signaling pathway as *MITF*). Importantly, two recently reported cases showed predominantly pink granular cytoplasm, making a distinction from conventional PEComas with *TSC1/2* mutations (or more rarely other molecular alterations) impossible on morphological grounds [20]. Generally, the main distinguishing feature is the frequent positivity for S100 protein, observed in five out of eight cases, three of which showed diffuse positivity. In contrast, S100 protein expression is relatively rare (and focal if present) in PEComas. Nevertheless, the striking similarity between *ACTIN::MITF*-rearranged tumors and PEComas is intriguing and raises a question whether *ACTIN::MITF* tumors represent a standalone entity, a distinct subtype of PEComa or whether *ACTIN::MITF* fusions may be shared by two different tumor types [4, 20].

## *EWSR1::SMAD3-*rearranged fibroblastic tumor

To date, 17 cases of this tumor have been documented in the English literature, affecting individuals across all age groups, with a slightly higher prevalence among women. The tumor shows a strong predilection for the acral location, particularly the lower limbs; one intraosseous case has been also described in the tibia [21]. It is a benign tumor which frequently recurs unless completely excised [22–24].

**Fig. 4:**
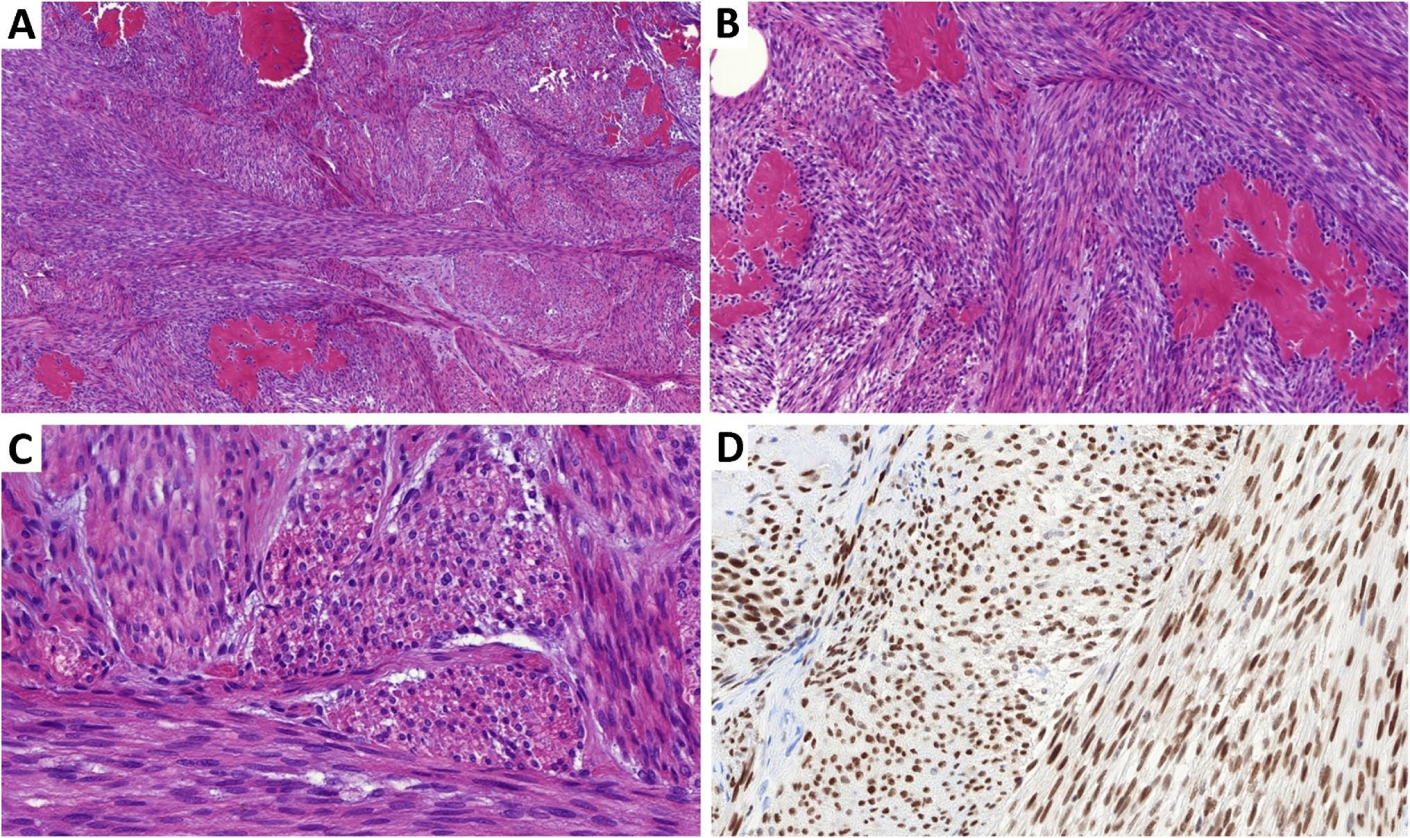
**A–C** *EWSR1::SMAD3*-rearranged tumor is usually consists of two components: densely packed cellular bundles of monomorphic bland spindle cells with prominent centrally located hyalinized stroma. **D** Uniform diffuse strong nuclear expression of ERG is characteristic

Histologically, the tumor typically consists of two components: densely packed cellular bundles of monomorphic bland spindle cells and prominent hyalinized stroma (Fig. 4A–C). Mitotic activity is usually absent. Immunohistochemically, a strong diffuse nuclear expression of ERG is observed, with intensity similar to that of capillary endothelium (Fig. 4D). In contrast, markers such as keratins, CD34, CD31, and others are typically negative; SMA may be occasionally weakly positive. In all cases, *EWSR1::SMAD3* fusion was present. *EWSR1* break-apart FISH probe can be used to confirm the diagnosis in challenging cases [23, 24].

In terms of differential diagnosis, it is crucial to distinguish this tumor from cellular spindle cell sarcomas such as monophasic synovial sarcoma, dermatofibrosarcoma protuberans, or low-grade fibromyxoid sarcoma. However, it is more commonly confused with benign spindle cell tumors such as myofibroma/myopericytoma, perineurioma, fibromatosis, and especially cellular fibrous histiocytoma. Almost all of these entities can be distinguished from *EWSR1::SMAD3*-rearranged fibroblastic tumor based on typical morphological and immunohistochemical features [24]. The only exception is cellular fibrous histiocytoma, which can closely resemble *EWSR1::SMAD3*-rearranged tumor morphologically and regularly show diffuse expression of ERG [25]. However, the intensity of ERG expression is typically weaker than that observed in the endothelium of surrounding vessels (or that observed in *EWSR1::SMAD3*-rearranged tumor) and comparison with these structures is diagnostically useful.

## Superficial CD34 + fibroblastic tumor

First characterized in 2014, this tumor typically affects adolescents or young adults and demonstrates a preference for the skin and subcutaneous tissue of the proximal limbs, particularly the thigh [26–30].

**Fig. 5:**
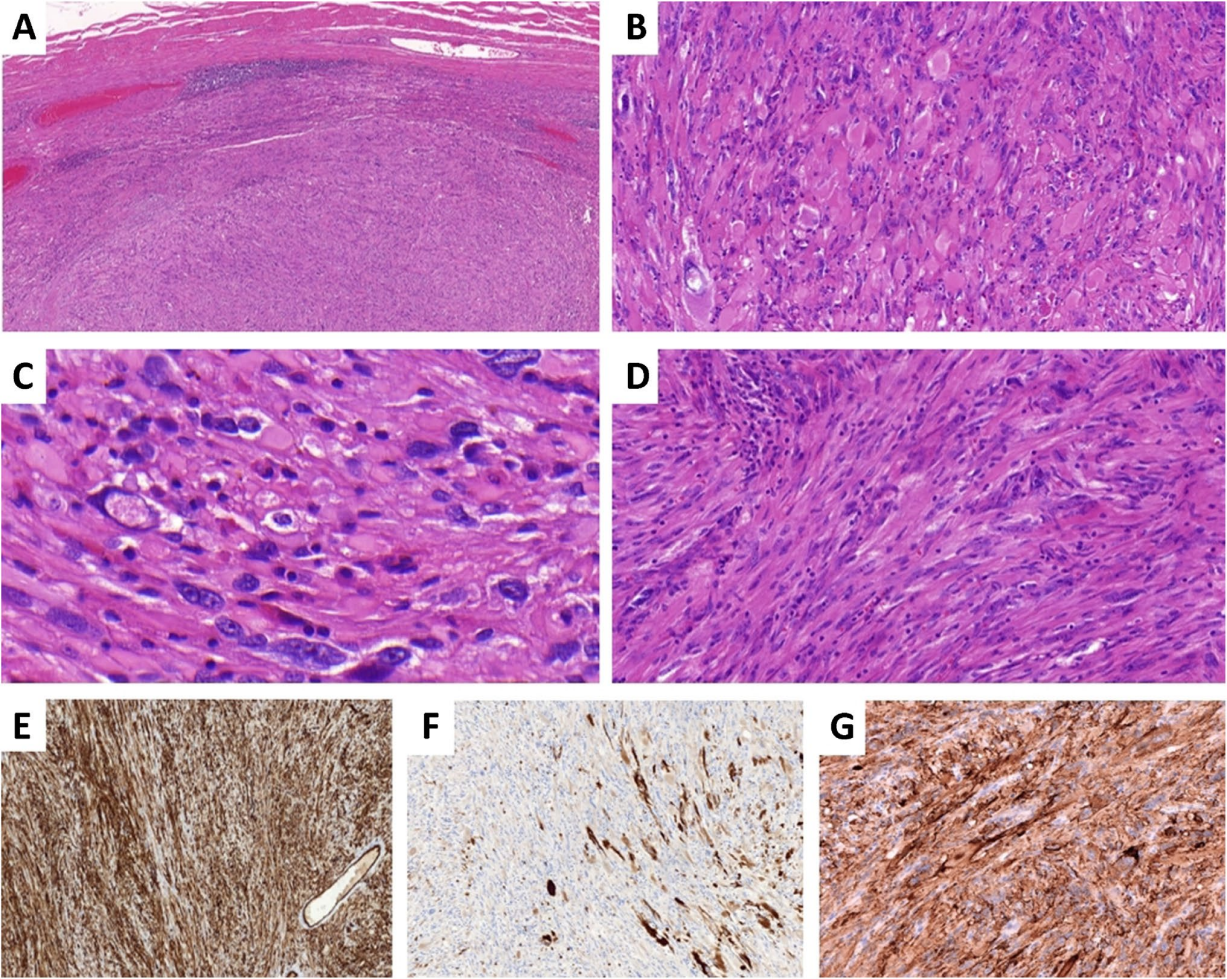
**A** Superficial CD34 + fibroblastic tumor is predominantly well-circumscribed, although small areas with infiltrative growth pattern may be present (not shown).** B**, **C** The tumor consists of sheets of epithelioid to spindle cells. The most striking features include abundant, markedly eosinophilic, granular-to-glassy cytoplasm and marked nuclear pleomorphism with frequent nuclear pseudoinclusions. Mitotic activity is generally low.** D** In some cases, spindle cell areas may be present and occasionally may predominate. **E** Superficial CD34 + fibroblastic tumor shows diffuse strong expression of CD34. **F** In approximately 70–80% of cases, tumor shows frequent but usually focal expression of cytokeratins. **G** SynCam3/CADM3 antibody is valuable diagnostic marker with high specificity and sensitivity

It commonly presents as a well-circumscribed tumor, although small infiltrative foci may be observed (Fig. 5A). Morphologically, the tumor consists of sheets of epithelioid cells (Fig. 5B, C) or spindle cell bundles (Fig. 5D). Perhaps the most striking features are the abundant, markedly eosinophilic, granular-to-glassy cytoplasm and marked nuclear pleomorphism with frequent nuclear pseudoinclusions (Fig. 5C). Despite this, the mitotic activity is generally low, and atypical mitoses are usually absent, although exceptions exist [26–30]. Rare cases may show collagen entrapment at the periphery, predominantly spindle cell morphology and somewhat less pronounced atypia, therefore mimicking atypical fibrous histiocytoma [31].

By definition, the tumor shows diffuse strong expression of CD34 (Fig. 5E), along with frequent but usually focal expression of cytokeratins (Fig. 5F) which is seen in approximately 70–80% of cases [26, 27, 30]. Nuclear and predominantly diffuse expression of WT-1 is observed in about two-thirds of cases (clone 6F-H2 against the N-terminus of WT1) [26]. A valuable diagnostic marker for this tumor is the SynCam3/CADM3 antibody (Fig. 5G), which is a highly specific and sensitive marker for these tumors based on the available studies [26, 29, 30] as well as in our experience. Approximately half of these tumors harbor *PRDM10* fusions with *CITED2*, *MED12*, *ARHGAP32*, or *RAD30* genes being the fusion partners [26, 29, 30]. There are no significant differences in clinical characteristics between cases with and without fusion.

Recognition of this tumor is crucial due to its deceptively malignant appearance, often leading to confusion with undifferentiated pleomorphic sarcoma. Despite worrisome morphological features, this lesion has an indolent clinical behavior with low recurrence rate and very rare regional lymph node metastasis [26–30]. Apart from undifferentiated pleomorphic sarcoma, the tumor is also frequently misdiagnosed as pleomorphic liposarcoma, dermatofibrosarcoma protuberans, or myxoinflammatory fibroblastic sarcoma. Correlating clinical features (superficial location, typically young patients), morphological appearance (characteristic cytoplasmic features, minimal mitotic activity), and immunophenotype is usually sufficient to differentiate these lesions.

## *NTRK*-rearranged spindle cell neoplasms

**Fig. 6:**
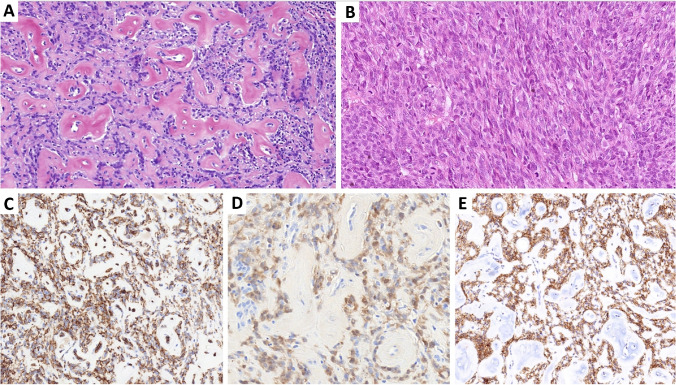
**A** A morphologically distinct subgroup of *NTRK*-rearranged spindle cell neoplasms are benign tumors with prominent stromal and perivascular hyalinization.** B** Sarcomatous transformation to fibrosarcoma is relatively common in this subgroup. Immunohistochemically, there is frequent co-expression of CD34 (**C**) and S100 protein (**D**) in this subgroup. **E** Expression of the CD30 antibody appears to be diagnostically useful

Soft tissue tumors with kinase gene alterations traditionally comprised a heterogeneous group of mesenchymal neoplasms which, besides others, included dermatofibrosarcoma protuberans, inflammatory myofibroblastic tumor, and infantile fibrosarcoma (IFS) [32]. While the canonical gene rearrangement of IFS, the *ETV6::NTRK3* fusion, was described more than two decades ago [33, 34], the recent widespread adoption of RNA sequencing (RNA-seq) has led to the description of several alternative gene rearrangements such as those of *NTRK1/2/3*, *BRAF*, *RET*, and others [32, 35–38]. The same technical breakthrough has also led to the emergence of a novel and not yet fully defined group of mesenchymal tumors with kinase gene alterations that morphologically and molecularly partially overlap with IFS [39, 40]. This group is currently included in the WHO classification of soft tissue and bone tumors as the emerging entity “*NTRK*-rearranged spindle cell neoplasms” (NTRK-SCN) [41, 42]. These tumors may arise at any anatomical site and affect both children and adults (although the younger age group more commonly). Morphologically, they include spindle cell tumors that form a continuous morphological spectrum which based on cellularity, degree of atypia, and mitotic activity can be roughly divided into three subgroups [39]. The first subgroup consists of clinically benign spindle cell lesions exhibiting low cellularity, minimal atypia, and very few mitoses (further referred to as the low-grade (LG) group), while the second contains morphologically high-grade lesions (high-grade (HG) group) that can follow an aggressive clinical course. In addition, some cases are difficult to assign to one of these categories as they fall somewhere in between these two extremes (intermediate-grade (IG) group). The LG group may exhibit one of several often co-occurring morphological patterns such as lipofibromatosis-like neural tumor pattern [43], prominent stromal and perivascular hyalinization pattern (Fig. 6A) [44, 45], myopericytic/haemangiopericytic pattern [46], and others [41, 47]. Tumors in the IG group may retain similar architecture as tumors from the LG group or they may vaguely resemble LG malignant peripheral nerve sheath tumors [41, 44]. Tumors from the HG group morphologically overlap with IFS, adult fibrosarcoma, and/or HG MPNST (Fig. 6B) [32, 41, 44]. Although some tumors display an uncharacteristic immunoprofile, many cases across the LG, IG, and HG spectrum exhibit immunohistochemical co-expression of CD34 (Fig. 6C) and S100 protein (Fig. 6D) [39, 41]. Additionally, the expression of the CD30 antibody (Fig. 6E) appears to be diagnostically useful as well [48]. Although panTRK immunohistochemistry is relatively sensitive for mesenchymal tumors with *NTRK1/2/3* fusions [49–52], it demonstrates imperfect specificity in skin and soft tissue mesenchymal tumors, as it can be positive in other neoplasms such as those with neural or myogenic differentiation [51, 52]. The molecular background of *NTRK*-SCN is characterized by various gene fusions and less often other alterations in kinase genes such as *NTRK1/2/3*, *BRAF*, *RAF1*, *RET*, *ALK*, or *MET* [41, 44, 53–61]. The list of these aberrations is broad and ever-expanding but in effect, they all lead to the constitutive activation of the MAP kinase signaling pathway [32].

Overall, NTRK-SCN may recur and metastasize in 7–26% and 5–20% of cases, respectively [39, 44, 62]. Accurate diagnosis of *NTRK*-SCN is clinically significant as complete excision can prevent recurrences and thus potential progression of LG lesions to more aggressive HG variants. Additionally, the availability of targeted therapy for advanced or metastatic cases of NTRK-SCN also makes recognition of this entity crucial [39].

Due to the broad morphological spectrum of these lesions, the differential diagnosis is diverse [41]. Tumors on the LG spectrum must be distinguished from other LG spindle cell neoplasms, such as lipofibromatosis, dermatofibrosarcoma protuberans, solitary fibrous tumor, or neurogenic tumors. The most challenging differential in cases from the HG spectrum are tumors with fibrosarcoma-like morphology such from malignant peripheral nerve sheath tumors or fibrosarcoma arising in dermatofibrosarcoma protuberans. In most cases, molecular studies are necessary for a confirmation of the diagnosis of *NTRK*-SCN.

## Other changes

Alterations in terminology and the incorporation of novel findings into existing lesions, particularly regarding their molecular genetic background were the most significant other changes. However, only those which we believe will have the highest impact on routine clinical practice will be mentioned.

### Changes in terminology

**Fig. 7:**
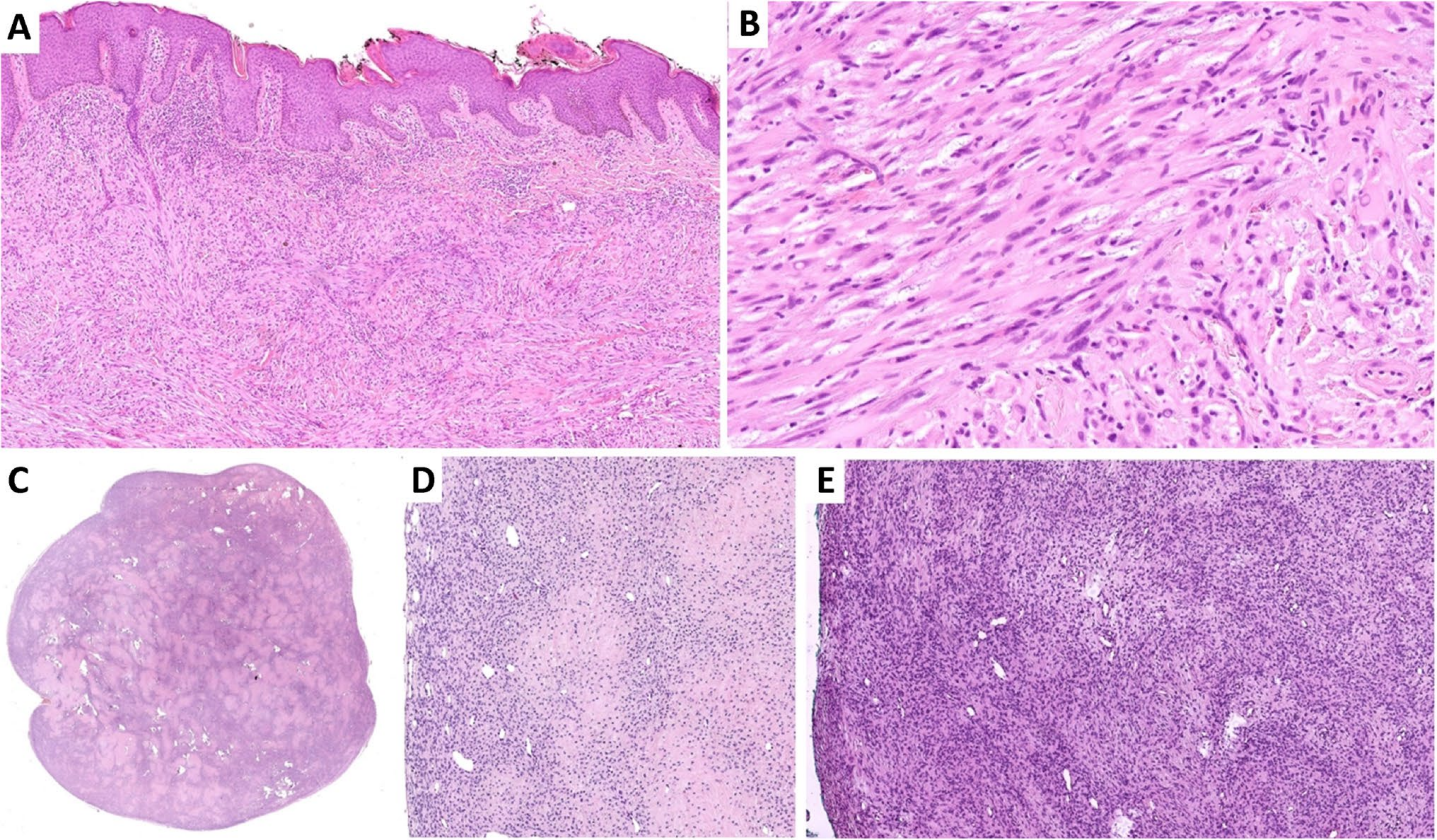
**A** Atypical intradermal smooth muscle neoplasia should exhibit a well-differentiated smooth muscle morphology. It should be limited to dermis with absent or minimal infiltration of the subcutaneous fat.** B** Morphology of this tumor is characterized by fascicles of predominantly uniform spindle cells with classic cigar-shaped nuclei and abundant eosinophilic cytoplasm.** C**, **D** DFSP with alternative *PDGFD* fusions often exhibit unusual clinicopathological features such as purely subcutaneous involvement or circumscribed nodular growth lacking the classic “honeycomb” pattern. Some cases may also show unusual morphological features. **E** A different case of *PDGFD*-rearranged DFSP showing sharply circumscribed border and presenting as a small subcutaneous nodule

One significant nomenclature change involves tumors previously known as cutaneous leiomyosarcoma. Due to favorable prognosis, the currently recommended term is atypical intradermal smooth muscle neoplasia (AISMN) [63, 64]. However, it is important to adhere to the definition: these tumors must be primarily located in the dermis (Fig. 7A) and have minimal infiltration of the subcutaneous tissue, the latter of which seem to portend a somewhat more aggressive behavior [64]. In addition, cases located predominantly in the subcutis (i.e., yet deeper than cases described above) which often arise from subcutaneous vessels have a much poorer prognosis that is comparable to the prognosis of deep soft tissue leiomyosarcomas and such cases should not be labeled as AISMN [65]. AISMN typically exhibit a well-differentiated smooth muscle morphology, characterized by fascicles of predominantly uniform spindle cells with classic cigar-shaped nuclei and abundant eosinophilic cytoplasm (Fig. 7B) [63, 64]. When a dermal tumor exhibits a poorly differentiated leiomyosarcoma morphology, a metastasis of a more deeply located soft tissue leiomyosarcoma needs to be excluded [66]. AISMN should also show smooth muscle immunoprofile with positivity for smooth muscle actin, desmin, and h-caldesmon. However, it is important to use the highly specific h-CD clone of h-caldesmon, as other available clones of this marker show very poor specificity [67].

Another significant terminological change involves the grouping of retiform hemangioendothelioma and papillary intralymphatic angioendothelioma under the collective name “hobnail hemangioendothelioma” since overlapping morphological features and clinical behavior made a reliable distinction of the two entities challenging as well as clinically irrelevant.

### Significant novel genetic findings

Although they occur in only a small percentage of cases, the description of alternative *PDGFD* rearrangements in cases of dermatofibrosarcoma protuberans lacking the canonical *COL1A1::PDGFB* fusion represents perhaps the most important new addition in this section due to the overall relatively high incidence of this neoplasm. Additionally, tumors with these novel fusions, namely *PDGFD::COL6A3*, *PDGFD::EMILIN2*, or *PDGFD::TNC*, often exhibit distinctive clinicopathologic features, such as preference for the thoracic region in women, subcutaneous involvement, and circumscribed nodular growth lacking the classic infiltrative “honeycomb” pattern (Fig. 7C, E) [68–72]. Some cases may also show unusual morphological features (Fig. 7C–E) [70–72]. However, their immunohistochemical features and prognosis remain similar to those of dermatofibrosarcoma protuberans with conventional molecular background.

**Fig. 8:**
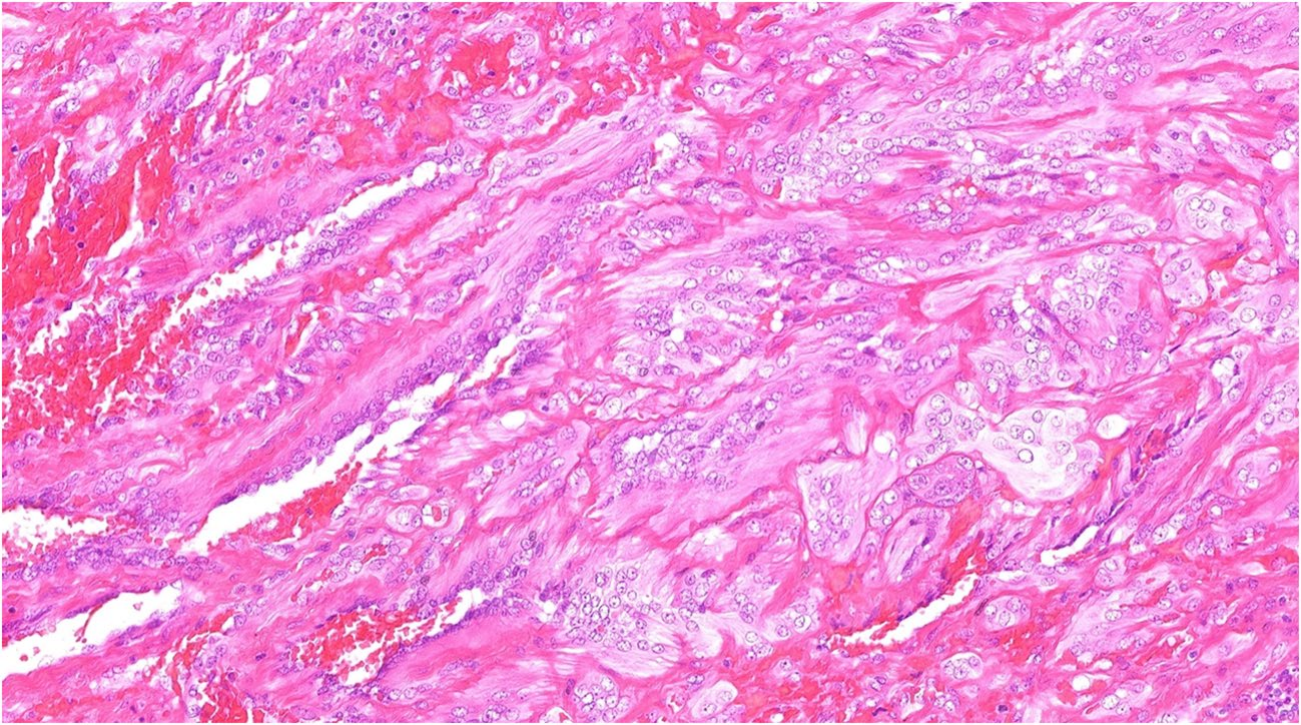
In neuroendocrine composite hemangioendothelioma, the vascular component, typically shows a retiform/hobnail hemangioendothelioma pattern admixed with nests of neuroendocrine/carcinoid-like cells

In the section on vascular neoplasms with intermediate malignant potential, a neuroendocrine variant of composite hemangioendothelioma has been incorporated into the respective chapter [73]. This variant is characterized by the combination of a vascular component typically resembling retiform/hobnail hemangioendothelioma, with nests of neuroendocrine cells (Fig. 8) [73, 74]. Notably, this variant shows more aggressive clinical behavior than conventional composite hemangioendothelioma and is associated with fusions of the *PTBP1::MAML2* or *EPC1::PCH2* genes; single cases also harbored *YAP1::FOXR1* and *ARID1B::MAML2* fusions [75]. Additionally, a frequent fusion of *YAP1* with the *MAML2* gene has been reported both in conventional composite hemangioendothelioma and in hobnail (retiform) hemangioendothelioma [76], highlighting the close relationship of these two entities.

## Declarations

The study was conducted following the rules set by the Faculty Hospital in Pilsen Ethics Committee. Informed consent was not required for the study
